# Reverse shoulder arthroplasty in revision surgery—Indications and results

**DOI:** 10.1371/journal.pone.0316440

**Published:** 2025-01-03

**Authors:** Patricia Bergert, Ralf Henkelmann, Pierre Hepp, Jan Theopold

**Affiliations:** Division of Arthroscopic and special Joint Surgery / Sports Injuries, Department of Orthopedics, Trauma and Plastic Surgery, University of Leipzig, Leipzig, Germany; Huashan Hospital Fudan University, CHINA

## Abstract

**Background:**

The number of reverse shoulder arthroplasty (RSA) procedures performed worldwide has increased over the last 10 years, with a corresponding increase in revision shoulder arthroplasty (SRSA). SRSA is often used for post-traumatic revision surgery in cases of infections and failure of anatomical prostheses. Data on outcomes with specific detail for each indication for the prosthetic solution as a secondary treatment are scarce, and inhomogeneous.

**Methods:**

The questionnaires were sent by mail to 65 patients who underwent SRSA between January 2014 and November 2023. Based on the indications for SRSA, patients were categorized into post-traumatic shoulder arthritis, humeral head necrosis, failed proximal humerus fractures, failed proximal humerus osteosynthesis, prostheses loosening, and infection groups.

**Results:**

Of the 65 patients included in the study, 39 completed the questionnaire, and the mean follow-up duration was 44 months (range, 12–104 months). The Constant score ranged from 28 points for all 6 groups (range, 38–66). The post-infection group showed the highest results, with 66 points (range, 24–90) on the Constant score; followed by 26 points (range, 49–6) points on the DASH score; and 0.90 (range, 0.763–1) on the EQ-5D-5L. Failed proximal humerus fractures presented the lowest scores: 38 points (range, 22–63) on the Constant score; 51 points (range, 73–30) points on the DASH score; and 0.61 (range, -0.496–1) on the EQ-5D-5L.

**Conclusions:**

No previous study has investigated the influence of indications on the clinical outcome of SRSA so circumstantial. In this study, the highest outcome scores were observed in the post-infection group, whereas the lowest scores were observed in the failed humerus fracture group. Our results underline the influence of the indication on the clinical outcome of SRSA.

## Background

Reverse shoulder arthroplasty (RSA) was initially designed to treat rotator cuff tear arthropathy in elderly patients with loss of active functionality [1, 2]. In the light of this success RSA has become a proven treatment option for cases of both degenerative and trauma-associated disease [3]. Furthermore, the scope of its indications has been extended to include revision arthroplasty (SRSA). Many surgeons use the SRSA as the most appropriate method of treatment for failed primary operations because the SRSA allows treatment of both soft-tissue and bony deficiencies [4, 5].

Various studies have demonstrated the use of RSA as a rescue procedure when faced with complex situations of the glenohumeral joint [6–9]. The difference in outcomes between SRSA and RSA applied as primary surgeries has been represented in the literature on repeated occasions [6, 10–12]. Although the SRSA is often associated with poorer post-operative outcomes than RSA, SRSA can be viewed in a positive light of balanced considerations, making it possible to restore or maintain the autonomy of affected patients [6, 7].

There are very few, if any, clinical trials on the subject of SRSA that focus on the indication for a revision surgery [6, 11, 12]. For this reason, the aim of this study was to determine the degree to which the clinical outcome is affected by the indication for revision surgery.

## Methods

Our retrospective, single-centre study carried out by post as a follow-up investigation included all patients treated with an SRSA between January 2014 and November 2023. The corresponding diagnosis codes (ICD-10) were used to identify cases in the hospital’s patient record system. All patients who received the shoulder arthroplasty as secondary therapy and were at least 18 years old were included in the study. After the study was conducted, individual patient data could no longer be identified and was analyzed anonymously.

The study was approved by the ethics committee of the University of Leipzig (AZ309/16-ek), and was carried out in accordance with the Declaration of Helsinki as well as the guidelines from the International Conference on Harmonization (Good Clinical Practice).

The patient cohort was divided into six groups based on their respective indications: post-traumatic shoulder arthritis (group 1), humeral head necrosis (group 2), failed proximal humerus fractures (group 3), failed proximal humerus osteosynthesis (group 4), prostheses loosening (group 5), and infections (group 6). Patients in groups 1,2, and 5 were included using the radiograph analysis. Group 1 showed a typical reduction in the joint space. Groups 3 and 4 were both identified by the Boileau classification. Group 6 included patients with infections after primary prostheses and after fracture treatment. In the event of an infection, the inserted prosthesis or osteosynthesis was removed. A vancomycin spacer was then implanted. The final treatment with an SRSA was only carried out after three punctures without the detection of a germ. All procedures were performed by three surgeons with extensive expertise in shoulder surgery. The used prostheses were the Delta Xtend Depuy, Global Unite (DePuy Synthes, Raynham, MA, U.S.), and the Aequalis PerFORM (Tornier SAS, Montbonnot-Saint-Martin, France). The Delta Xtend Depuy and Global Unite have an inclination angle of 155°, the Aequalis PerFORM has an inclination angle of 135°. No lateralization took place. Outcomes were evaluated using the Constant, DASH and EQ-5D-5L index scores.

We investigated subjective patient satisfaction, perception of pain, functionality, and possible range of motion in the glenohumeral joint following SRSA.

Three different scores were used for the analysis: Constant-Score; DASH-Score; EQ-5D-5L. Forms were sent out by post due to the ongoing COVID-19 pandemic, and patients completed the forms independently. If patients underwent multiple revisions, they always received the questionnaire after the last procedure.

Statistical analysis was conducted using the IBM SPSS Statistics 28 software (SPSS Inc., Chicago, IL, USA). Because of the study’s explorative nature and the expected small sample size, we performed a descriptive statistical test.

## Results

65 SRSA procedures were carried out between January 2014 and November 2023. All of these scores were collected for 39 patients (60%; 23 women; 16 men). 8 patients (12%) were deceased by the time of the examination. 18 (28%) could not be contacted.

**Fig 1:**
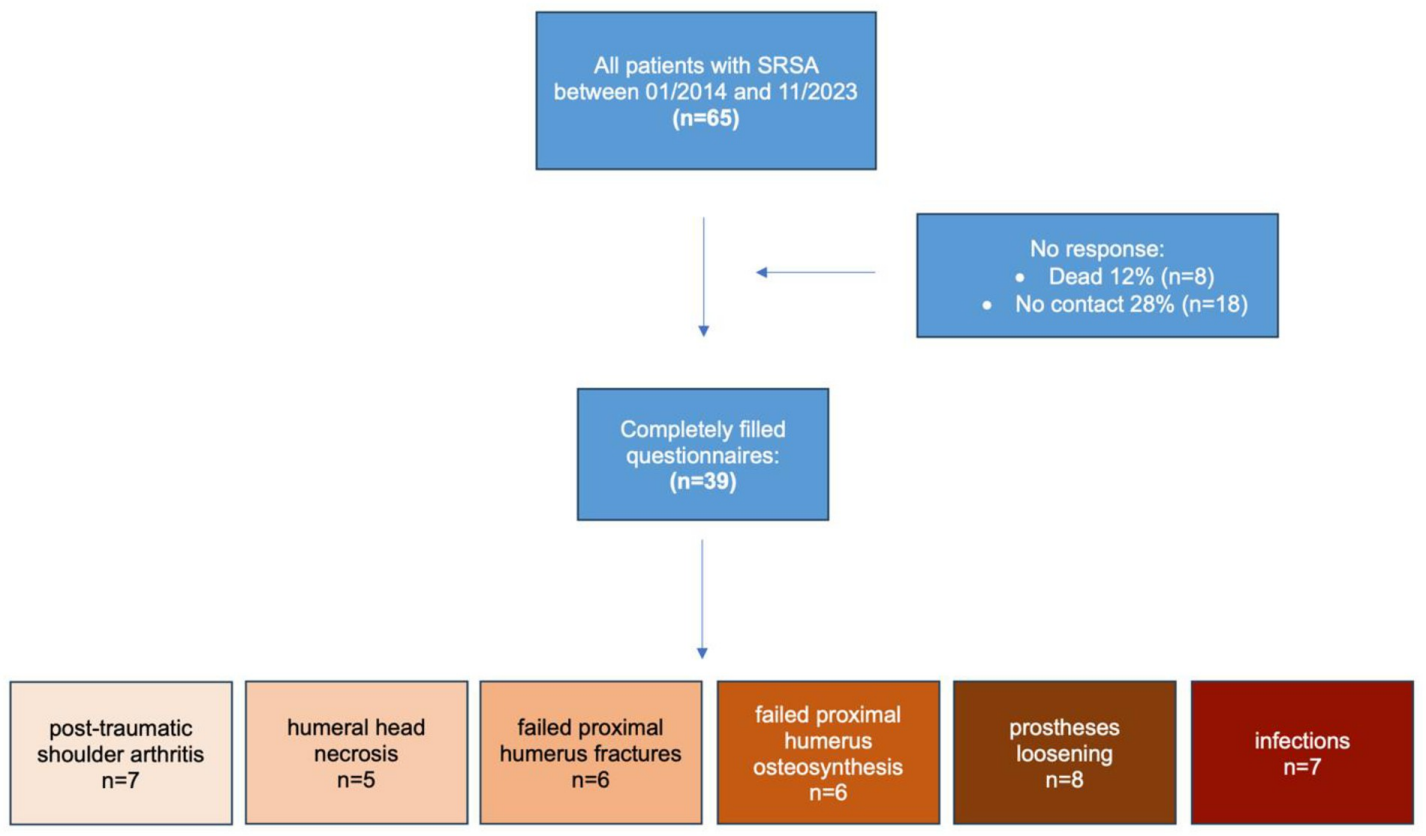
Flowchart visualization of the process of patient selection.

We divided the patient population into 6 groups according to the indication (Fig 1): post-traumatic shoulder arthritis (group 1) (n = 7; 18%; mean age, 68.7); humeral head necrosis (group 2) (n = 5; 13%; mean age, 67.2); failed proximal humerus fractures (group 3) (n = 6; 15%; mean age, 64.5); failed proximal humerus osteosynthesis (group 4) (n = 6; 15%; mean age, 72.0); prosthesis loosening (group 5) (n = 8; 21%; mean age, 68.75); and infections (group 6) (n = 7; 18%; mean age, 68.7).

The average age at the time of surgery was 68.03 years (range, 48–86 years). The average follow-up time was 44 months (range, 12–104 months). On average, the entire cohort achieved a Constant score of 51 (range, 19–91); a DASH score of 41 (range, 87–6); and an EQ-5D-5L of 0.75 (range, -0.496–1).

Group 6 showed the highest results, at 66 points (range, 24–90) on the Constant score; 26 points (range, 49–6) points on the DASH score; and 0.90 (range, 0.763–1) on the EQ-5D-5L. Group 5 achieved 62 points (range, 33–84) on the Constant score; 31 (range, 49–11) on the DASH score; and a result of 0.81 (range, 0.697–0.943) on the EQ-5D-5L.

**Fig 2:**
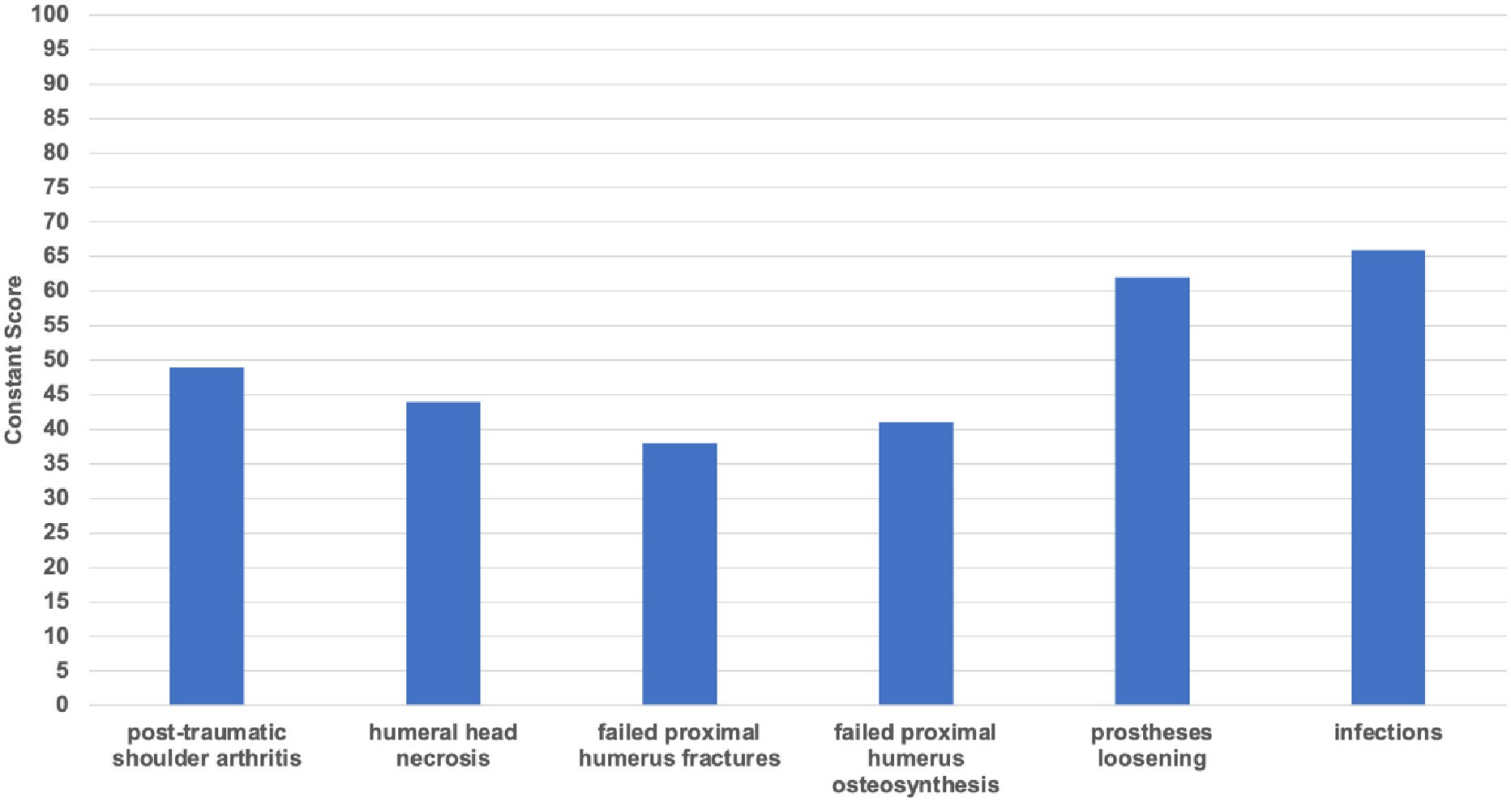
Clinical outcome: Constant score of all six groups at least twelve months post-operatively.

**Fig 3:**
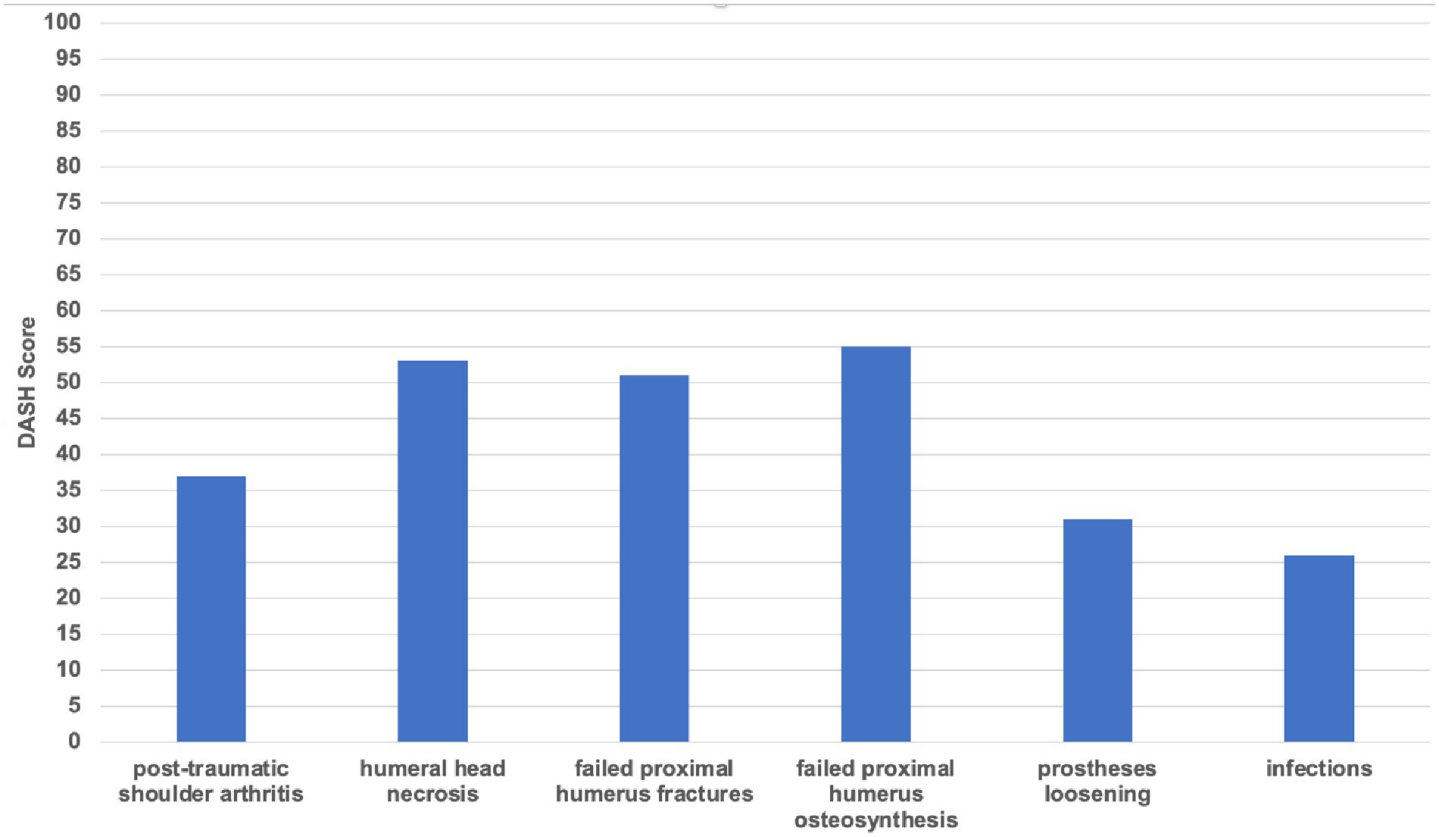
Clinical outcome: DASH score of all six groups at least twelve months post-operatively.

**Fig 4:**
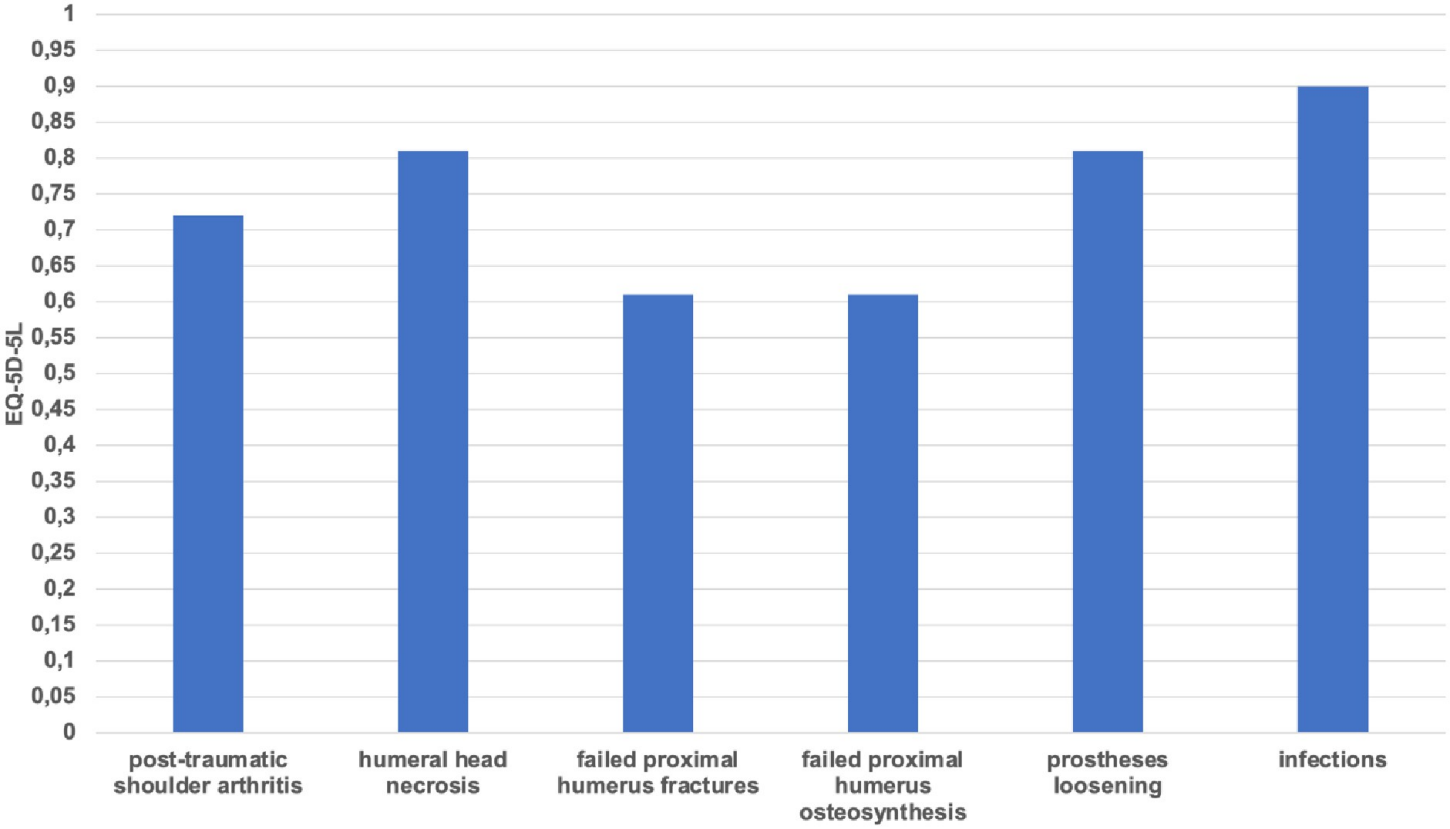
Clinical outcome: EQ-5D-5L score of all six groups at least twelve months post-operatively.

Group 1 achieved 49 points (range, 19–78) on the Constant score; 37 points (range, 81–8) points on the DASH score; and 0.72 (range, -0.176–1) on the EQ-5D-5L. Group 2 scored 44 points (range, 26–57) on the Constant score; 54 (range, 87–28) on the DASH score; and a result of 0.81 (range, 0.743–0.881) on the EQ-5D-5L. Group 4 achieved 41 points (range, 20–91) on the Constant score; 55 (range, 85–7) on the DASH score; and a result of 0.61 (range, 0.028–1) on the EQ-5D-5L. Group 3 showed the lowest results, at 38 points (range, 22–63) on the Constant score; followed by 51 points (range, 73–30) points on the DASH score; and 0.61 (range, -0.496–1) on the EQ-5D-5L. All results are illustrated in figures (Figs 2–4).

The differences between the six groups due to the indication were not significant (P Constant Score = .196; P DASH Score = .16; P EQ-5D-5L = .683).

## Discussion

The results from our study have demonstrated that indication is the decisive criterion regarding clinical outcome of SRSA. There was a difference of 28 points in the Constant score between the different groups with the highest and lowest results, which shows the great relevance of the indication with respect to the post-operative result.

We were able to evaluate 60% of patients who had an SRSA. A response rate greater than 50% in study carried out by post as a follow-up investigation ensures representative results [13, 14].

It has been reported that males have a higher rate of revision than females [15]. In our study we were unable to prove this. Our cohort included more women who underwent revision surgery.

The entire cohort achieved an average Constant score of 51 points, with a mean follow-up time of 44 months. This value correlates with results from the literature. Flury et al. (2011) reported a similar mean Constant score for patients following SRSA, at 46 months post-operatively. The Constant score for their patients was slightly higher, but this could be due to the fact that significantly fewer patients were included in their study [9].

There are also descriptions in the literature of the lack of studies investigating the changeover to the SRSA [12]. Furthermore, several authors have concluded that the indication for SRSA is decisive with regards to the post-operative clinical outcome [11, 16].

It has already been established that the time elapsed between the index surgery and revision has no influence on the subsequent clinical outcomes [7]. For this reason, we decided not to take this factor into account, and to focus only on the indications.

There are descriptions in the literature arguing that instability or loss of function of a prosthesis are the most common reasons for revision [12, 15]. This is also true for our cohort, with loosening of prostheses representing the largest group, at 21%. Moen et al. concluded in their study that the indication of prosthesis loosening leads to the worst functional results [17]. We were not able to demonstrate this in our study: with a Constant score of 62 points, these patients achieved the second highest result.

Group 6 reached the highest scores. This fact is supported by the study of Lo et al. They assumed that the implantation of a SRSA is an effective treatment for infections, maybe the established gold standard [18].

The lowest scores were reached for the SRSAs following failed treatment for fracture or fracture sequelae (Groups 1,2,3 and 4). This finding is consistent with the results of Gauci et al. According to this study, surgeons should be aware that failed fracture treatment is a risk factor for SRSA [4].

Gauci et al. reported that the incidence of humeral complications is increasing. In our study, we can confirm this, 24 of 39 patients (Groups 1, 2, 3, 4) were revised with a SRSA following a humeral complication [4]. Due to the complexity of failed fracture treatment, Maccagnano et al. suggest to evaluate before surgical choice not only anatomical parameters but also patient psychological profile. The implantation of SRSA should always be weighed up critically [19]. It is suggested that the implantation of SRSA is an advantage over ORIF in the treatment of displaced failed treated humerus fractures [20].

There is a broad agreement in the literature that primary RSA performs better than SRSA [6]. However, Shannon et al. showed that the use of the SRSA as a treatment for failed osteosynthesis results in the same clinical outcome and range of motion. This underlines once again the relevance of the SRSA as a revision procedure [21].

In summary, the implantation of a RSA as a revision procedure represents a challenging procedure for a complex group of patients who require a sufficient level of expertise, and this procedure should be carried out in a centre that performs this procedure regularly [10, 22].

No previous study has examined the outcomes of SRSA in such a differentiated manner in relation to the underlying indication, which is why there are few, if any, comparative data regarding the different aetiologies.

Overall, the results from the SRSA should be investigated in prospective studies with regard to their indications.

Our study also has several limitations. The retrospective design leads to higher case losses and data records cannot be corrected. This resulted in an effect called bias, but to prevent this effect we only included completely filled questionnaires [23].

Another limitation is the lack of preoperative scores. However, the focus of our study was on the effect of the indication on the clinical outcome and not on the improvement of the surgical intervention.

The analysis of tuberosity healing in Group 5 would undoubtedly have been of interest. However, due to the small sample size and the lack of statistical significance, further studies with larger cohorts are needed to draw meaningful conclusions. The realization as a self-assessment-survey is not really a limitation, because patients always evaluate their functionality less better than a medical doctor [24].

## Conclusions

In conclusion, in the present study, the six groups present very different results. This underlines the influence of the indication on the clinical outcome. Infections reach the highest scores. Failed proximal humerus fractures show the lowest scores. We must assume that the indication is the decisive criterion for the clinical outcome.

## Abbreviations

RSA: Reverse shoulder arthroplasty
SRSA: Secondary reverse shoulder arthroplasty

## References

[1] Boileau P, Melis B, Duperron D, Moineau G, Rumian AP, Han Y (2013) Revision surgery of reverse shoulder arthroplasty. Journal of Shoulder and Elbow Surgery 22:1359–1370. doi: 10.1016/j.jse.2013.02.004 23706884

[2] Boileau P (2016) Complications and revision of reverse total shoulder arthroplasty. Orthopaedics & Traumatology: Surgery & Research 102:S33–S43. doi: 10.1016/j.otsr.2015.06.031 26879334

[3] Georgoulas P, Fiska A, Ververidis A, Drosos GI, Perikleous E, Tilkeridis K (2021) Reverse Shoulder Arthroplasty, Deltopectoral Approach vs. Anterosuperior Approach: An Overview of the Literature. Front Surg 8:721054. doi: 10.3389/fsurg.2021.721054 34869550 PMC8636448

[4] Gauci M-O, Cavalier M, Gonzalez J-F, Holzer N, Baring T, Walch G, et al. (2020) Revision of failed shoulder arthroplasty: epidemiology, etiology, and surgical options. Journal of Shoulder and Elbow Surgery 29:541–549. doi: 10.1016/j.jse.2019.07.034 31594726

[5] Kokkalis ZT, Bavelou A, Papanikos E, Kalavrytinos D, Panagopoulos A (2022) Reverse Shoulder Arthroplasty for Failed Operative Treatment of Proximal Humeral Fractures. J Shoulder Elb Arthroplast 6:24715492221090742. doi: 10.1177/24715492221090742 35669618 PMC9163725

[6] Bois AJ, Knight P, Alhojailan K, Bohsali KI (2020) Clinical outcomes and complications of reverse shoulder arthroplasty used for failed prior shoulder surgery: a systematic review and meta-analysis. JSES Int 4:156–168. doi: 10.1016/j.jses.2019.10.108 32195479 PMC7075779

[7] Schwarz A, Hohenberger G, Sauerschnig M, Niks M, Lipnik G, Mattiassich G, et al. (2021) Effectiveness of reverse total shoulder arthroplasty for primary and secondary fracture care: mid-term outcomes in a single-centre experience. BMC Musculoskelet Disord 22:48. doi: 10.1186/s12891-020-03903-0 33419418 PMC7792308

[8] Boelch SP, Streck LE, Plumhoff P, Konrads C, Gohlke F, Rueckl K (2020) Infection control and outcome of staged reverse shoulder arthroplasty for the management of shoulder infections. JSES Int 4:959–963. doi: 10.1016/j.jseint.2020.08.012 33345240 PMC7738577

[9] Flury MP, Frey P, Goldhahn J, Schwyzer H-K, Simmen BR (2011) Reverse shoulder arthroplasty as a salvage procedure for failed conventional shoulder replacement due to cuff failure—midterm results. Int Orthop 35:53–60. doi: 10.1007/s00264-010-0990-z 20229269 PMC3014498

[10] Chalmers PN, Boileau P, Romeo AA, Tashjian RZ (2019) Revision Reverse Shoulder Arthroplasty. J Am Acad Orthop Surg 27:426–436. doi: 10.5435/JAAOS-D-17-00535 31170096

[11] Stechel A, Fuhrmann U, Irlenbusch L, Rott O, Irlenbusch U (2010) Reversed shoulder arthroplasty in cuff tear arthritis, fracture sequelae, and revision arthroplasty: Outcome in 59 patients followed for 2–7 years. Acta Orthopaedica 81:367–372. 20450427 10.3109/17453674.2010.487242 PMC2876841

[12] Merolla G, Wagner E, Sperling JW, Paladini P, Fabbri E, Porcellini G (2018) Revision of failed shoulder hemiarthroplasty to reverse total arthroplasty: analysis of 157 revision implants. J Shoulder Elbow Surg 27:75–81. doi: 10.1016/j.jse.2017.06.038 28751094

[13] Parekh AD, Bates JE, Amdur RJ (2020) Response Rate and Nonresponse Bias in Oncology Survey Studies. American Journal of Clinical Oncology 43:229. doi: 10.1097/COC.0000000000000665 31972569

[14] Menold N (2016) Postal SurveysPostal Surveys. GESIS Survey Guidelines.

[15] 2021 Annual Report Patient Presentation for Surgeons—SHOULDERS.pdf.

[16] Boileau P, Gonzalez J-F, Chuinard C, Bicknell R, Walch G (2009) Reverse total shoulder arthroplasty after failed rotator cuff surgery. Journal of Shoulder and Elbow Surgery 18:600–606. doi: 10.1016/j.jse.2009.03.011 19481959

[17] Moen P, Patel JS, Simon P, Christmas KN, Haidamous G, Frankle MA (2023) Humeral loosening in reverse shoulder arthroplasty: an analysis of 2,342 cases. J Shoulder Elbow Surg 32:S53–S59. doi: 10.1016/j.jse.2023.02.006 36822498

[18] Lo EY, Ouseph A, Badejo M, Lund J, Bettacchi C, Garofalo R, et al. (2023) Success of staged revision reverse total shoulder arthroplasty in eradication of periprosthetic joint infection. Journal of Shoulder and Elbow Surgery 32:625–635. doi: 10.1016/j.jse.2022.09.006 36243299

[19] Maccagnano G, Solarino G, Pesce V, Vicenti G, Coviello M, Nappi VS, et al. (2022) Plate vs reverse shoulder arthroplasty for proximal humeral fractures: The psychological health influence the choice of device? World J Orthop 13:297–306. doi: 10.5312/wjo.v13.i3.297 35317248 PMC8935332

[20] Fraser AN, Bjørdal J, Wagle TM, et al (2020) Reverse Shoulder Arthroplasty Is Superior to Plate Fixation at 2 Years for Displaced Proximal Humeral Fractures in the Elderly: A Multicenter Randomized Controlled Trial. J Bone Joint Surg Am 102:477–485. doi: 10.2106/JBJS.19.01071 31977825 PMC7508281

[21] Shannon SF, Wagner ER, Houdek MT, Cross WW, Sánchez-Sotelo J (2016) Reverse shoulder arthroplasty for proximal humeral fractures: outcomes comparing primary reverse arthroplasty for fracture versus reverse arthroplasty after failed osteosynthesis. Journal of Shoulder and Elbow Surgery 25:1655–1660. doi: 10.1016/j.jse.2016.02.012 27101774

[22] Holton J, Yousri T, Arealis G, Levy O (2017) The role of reverse shoulder arthroplasty in management of proximal humerus fractures with fracture sequelae: a systematic review of the literature. Orthopedic Reviews. doi: 10.4081/or.2017.6977 28286622 PMC5337776

[23] Lenk C, Duttge G, Fangerau H (eds) (2014) Handbuch Ethik und Recht der Forschung am Menschen.

[24] Boehm D, Wollmerstedt N, Doesch M, Handwerker M, Mehling E, Gohlke F (2004) Entwicklung eines Fragebogens basierend auf dem Constant-Murely-Score zur Selbstevaluation der Schulterfunktion durch den Patienten. Unfallchirurg 107:397–402. doi: 10.1007/s00113-004-0757-3 15060778

